# Complexity-based graph convolutional neural network for epilepsy diagnosis in normal, acute, and chronic stages

**DOI:** 10.3389/fncom.2023.1211096

**Published:** 2023-09-29

**Authors:** Shiming Zheng, Xiaopei Zhang, Panpan Song, Yue Hu, Xi Gong, Xiaoling Peng

**Affiliations:** ^1^Guangdong Provincial Key Laboratory of Interdisciplinary Research and Application for Data Science, BNU-HKBU United International College, Zhuhai, China; ^2^Department of Neurology, Children's Hospital of Chongqing Medical University, Chongqing, China

**Keywords:** EEG complexity measures, entropy, graph convolutional neural network, epilepsy diagnosis, chronic stage

## Abstract

**Introduction:**

The automatic precision detection technology based on electroencephalography (EEG) is essential in epilepsy studies. It can provide objective proof for epilepsy diagnosis, treatment, and evaluation, thus helping doctors improve treatment efficiency. At present, the normal and acute phases of epilepsy can be well identified through EEG analysis, but distinguishing between the normal and chronic phases is still tricky.

**Methods:**

In this paper, five popular complexity indicators of EEG signal, including approximate entropy, sample entropy, permutation entropy, fuzzy entropy and Kolmogorov complexity, are computed from rat hippocampi to characterize the normal, acute, and chronic phases during epileptogenesis. Results of one-way ANOVA and principal component analysis both show that utilizing complexity features, we are able to easily identify differences between normal, acute, and chronic phases. We also propose an innovative framework for epilepsy detection based on graph convolutional neural network (GCNN) using multi-channel EEG complexity as input.

**Results:**

Combining information of five complexity measures at eight channels, our GCNN model demonstrate superior ability in recognizing the normal, acute, and chronic phases. Experiments results show that our GCNN model reached the high prediction accuracy above 98% and F1 score above 97% among these three phases for each individual rat.

**Discussion:**

Our research practice based on real data shows that EEG complexity characteristics are of great significance for recognizing different stages of epilepsy.

## 1. Introduction

Epilepsy is a neurological disorder defined as a transient occurrence of clinical features produced by abnormal excessive or synchronous neuronal (Fisher et al., 2005). Worldwide, more than 50 million people have epilepsy, affecting humans of all ages, ethnicity, and society. It has been classified as one of the most highly challenging neural psychiatric diseases that the World Health Organization (WHO) focuses on prevention and treatment (Saxena and Li, 2017). Epilepsy is characterized by recurrent seizures caused by abnormal discharge of brain neurons and an ongoing predisposition to recurrent seizures. The patients with epilepsy mainly include those with reflex seizures and those with more than one unprovoked seizure after 24 h. In particular, compared to the general population, the probability of having recurrent seizures in the next 10 years for epileptic patients who have had a single seizure is at least 60% (Fisher et al., 2005). Therefore, the diagnosis and treatment of epilepsy are of great significance for humans, while accurate prediction of epileptic seizures is crucial for achieving precision treatments in epilepsy. The rat pilocarpine (PILO) model of temporal lobe epilepsy (TLE) is an animal model in which central cholinergic receptors are activated to induce seizures by pilocarpine, a post-ganglionic cholinergic drug that can produce quasi-cholinergic effects by directly exciting M-cholinergic receptors (Song et al., 2016). Since the damage and indications of the rat PILO model are comparable to those of human TLE, it is a widely used animal epilepsy model of TLE. This model exhibits three important phases (Song et al., 2016): (1) the normal phase—1 day before status epilepticus (SE), (2) the acute phase—the duration of SE and 6–24 h after SE, and (3) the chronic phase—marked by occurrences of spontaneous recurrent seizures (SRS) after SE.

As one of the most potent and economical tools to record and monitor the brain's electrical activity, in recent years, electroencephalogram (EEG) analysis has become a hot topic in epilepsy diagnosis, and related studies for both doctors and researchers (Karlócai et al., 2011). Analyzing EEG recordings can provide an objective reference for diagnosing epilepsy-related diseases, such as the identification, prediction, focus location, or treatment evaluation of epilepsy (Karlócai et al., 2011). Various features extracted from EEG signals play essential roles in disease diagnosis as they can help researchers to describe the characteristics and mechanism of epileptic seizures. Basically, EEG signal features are divided into four categories. Time-domain features analyze how signal changes with time (Srinivasan et al., 2005; Sharmila and Geethanjali, 2018; Wei et al., 2019), frequency-domain features depict how signal lies within each frequency band (Srinivasan et al., 2005; Faust et al., 2010; Wen and Zhang, 2017), time-frequency domain features are characteristics consider both time and frequency domain (Tzallas et al., 2009; Wang et al., 2017), while nonlinear features regard the brain as a system to describe its complexity and the amount of information (Yuan et al., 2011; Li et al., 2017; Wang et al., 2017). Many previous studies have made significant progress in epilepsy detection based on one or more of these EEG signal features (Boonyakitanont et al., 2020). Since EEG signal shows non-stationary and nonlinear dynamic behavior when measuring the electrical activity of a brain (Natarajan et al., 2004), EEG signal features based on nonlinear dynamic properties may be better than the other three types of features in mining and detecting the regular changes of EEG in different stages of epileptogenesis. Recently, more and more researchers treated the dynamic changes of brain activity as a complex nonlinear system to study their complexity. Thus, some nonlinear complexity measures, especially various entropy indices, have attracted the great attention of researchers through their outperformance in characterizing EEG signals by quantifying the complexity and amount of information (Liang et al., 2015).

Most early studies achieved good performance for applying complexity measures and one or more classifiers to distinguish different stages of epilepsy by analyzing EEG signals. Sharma et al. (2014) built epileptic seizure detection models based on four complexity measures, including Shannon entropy, Renyi entropy, approximate entropy (ApEn), and sample entropy (SampEn), to classify the EEG signals during focal and non-focal epilepsy and achieved 87% accuracy by the least squares support vector machine (LS-SVM) classifier. To achieve auto-detection of focal and non-focal EEG recordings, Arunkumar et al. (2017) yielded the highest accuracy of 98% by feeding five different entropy features to the non-nested generalized exemplars (NNge) classifier after comparing with other four different classifiers, including naıve bayes classifier (NBC), radial basis function (RBF), support vector machines (SVM), and *k* nearest neighbor (KNN). Xiang et al. (2015) trained SVM using fuzzy entropy (FuzzEn) to detect epileptic seizures from normal groups and reached a detection rate of 98.31 and 100% on two different datasets, respectively.

However, most of these notable results were obtained from distinguishing epileptic EEG signals in the acute stage of epilepsy from normal. The study on EEG characteristics in the chronic stage has seldom been mentioned. Due to the fact that epilepsy patients are mostly in the chronic phase rather than the acute phase, identifying the chronic phase of epilepsy is particularly important for the timely diagnosis and treatment of epilepsy. It is beneficial to study and predict the chronic phase of epilepsy: (1) the pathophysiological mechanism of epilepsy and the effects and side effects of long-term medication in epileptic patients can be better understood; (2) and chronic seizures of epilepsy patients can be intervened and treated in advance. Hence, the primary motivation behind this work is to clarify the role of the complexity measures of EEG signals during acute and chronic seizures from normal groups. Further, it has been observed that most studies used traditional machine learning algorithms, such as SVM, Decision Tree, and KNN, to implement the classification tasks. Due to the simplistic structure of these conventional machine learning algorithms, only a single channel of EEG signals can be considered in the classification tasks. Nevertheless, multi-channel EEG is widely used for diagnosis and therapy in clinical practice because brain diseases are rarely limited to a specific region (Bullmore and Sporns, 2009). This prompted us to consider an advanced classifier that can integrate multi-channel EEG for epileptic detection.

Graph convolutional neural network (GCNN) is a deep neural network classification model capable of handling multichannel EEG signal analysis (Craley et al., 2022). It is an improvement of convolutional neural networks (CNN) and can preserve richer connection information than 2D or 3D matrices by considering EEG signals to be nodes in a topological graph and representing the relationships between them using edges (Lian et al., 2020). GCNN can describe the internal relationship between different graph's nodes, therefore providing a way to explore the relationship among multiple EEG channels in the EEG-based classification (Song et al., 2018). Thus, in recent years, GCNN has been applied and made an enormous impact on EEG-based recognition, including emotion recognition (Zhang et al., 2019), neurological disease diagnosis (Wagh and Varatharajah, 2020), sleep stage classification (Jia et al., 2020), epilepsy diagnosis (Covert et al., 2019; Li and Jung, 2021), and brain motor imagery (Hou et al., 2022).

In this paper, we developed an automatic epileptic detection system via GCNN using five complexity measures of EEG, including approximate entropy, sample entropy, permutation entropy, fuzzy entropy, and Kolmogorov complexity to monitor dynamic changes and distinguish EEG recordings among normal, acute, and chronic stage of epilepsy. Statistically significant indicators are useful in indicating the difference between chronic and normal stages, prompting doctors to intervene in advance.

## 2. Materials and methods

### 2.1. EEG recordings

The experimental data used in this paper was from a previous study (Song et al., 2016), in which the rat PILO model of TLE is used in this experiment (Song et al., 2016). In particular, the subject rats were injected with pilocarpine to induce seizures and were stopped by utilizing diazepam. The EEG signals were recorded during the experiment by drilling holes in the skull at specific locations and implanting microelectrodes. The coordinates for particular sites of interest in the hippocampus in our study are shown in Table 1.

**Table 1:** Electrode coordinates for areas of interest in the rat PILO model of TLE during epileptogenesis.

**Names of parts**	**Coordinates**
Cornu ammonis 1 (CA1)	AP: 3.3–3.7 mm from Bregma, ML: 2.0–3.0 mm, and DV: 3.0–3.5 mm from the surface of neocortex
Cornu ammonis 3 (CA3)	AP: 3.3 mm, ML: 3.5–3.7 mm, and DV: 3.0–3.5 mm
The surface of neocortex of the bilateral parietal lobe (Reference Electrode)	AP: 7.0 mm, ML: 6.0 mm
Dentate gyrus (DG)	AP: 5.6 mm, ML: 4.0 mm, and DV: 6.0 mm

**Figure 1:**
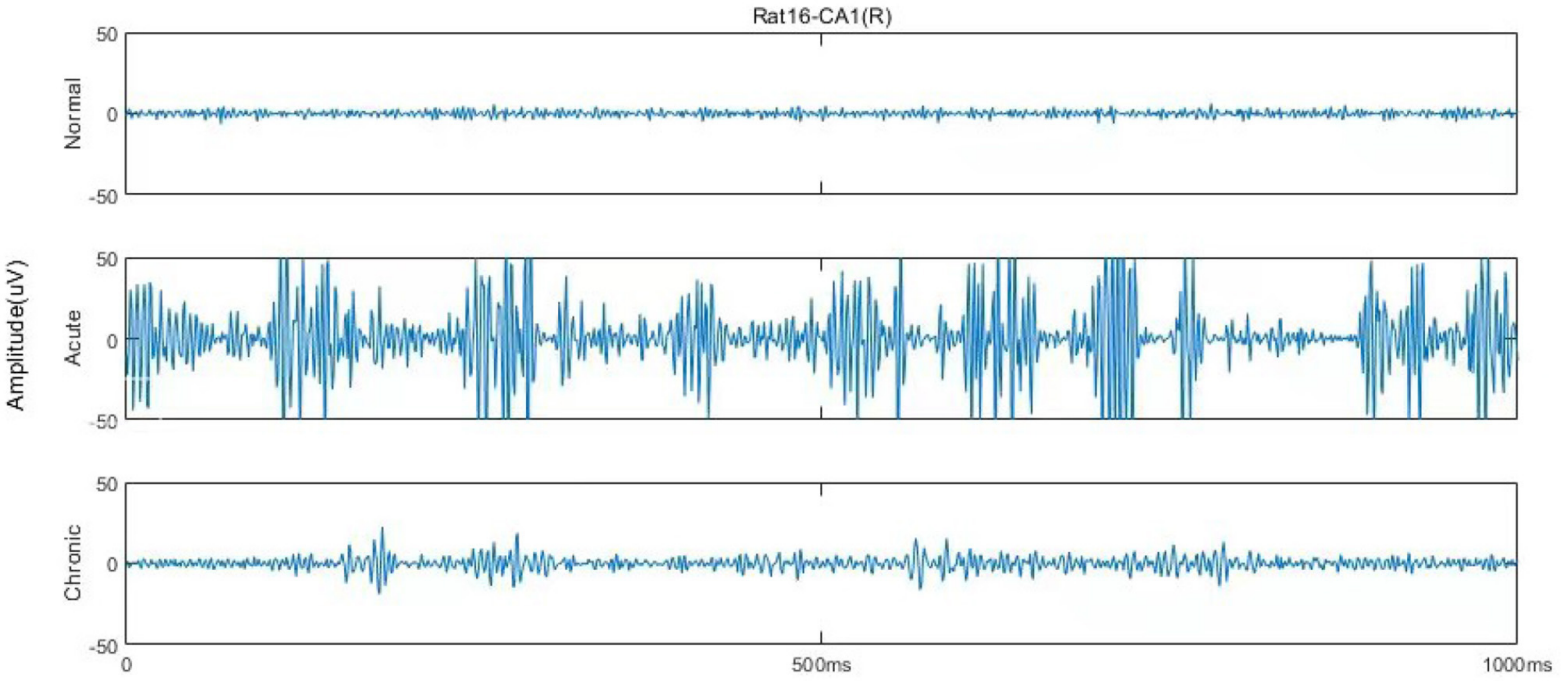
One second EEG waveforms in normal, acute, and chronic phases from rat no.16 in channel CA1(R), 250–500 Hz.

According to Song et al. (2016), each EEG recording has around 600,000 sampling points (10 min), and the original dataset could be mainly divided into six stages, including normal (1 day before SE), pre-seizure (30, 20, and 10 min before SE), acute [10 min after SE, 10 min before, and after utilizing diazepam (i.e., DZP injection)], stable (1, 2, and 3 h after the diazepam), latent (1, 3, and 7 days after SE), chronic (7, 14, and 28 days after SE) stages. Figure 1 describes and compares the 1 s waveforms (250–500 Hz) selected randomly from normal, acute, and chronic phases for representative rat (no.16) in channel CA1(R). Intuitively looking from Figure 1, the EEG of the acute phase is far from that of the normal and the chronic phases, with much wider amplitude and some typical waveform, while the difference between the normal phase and the chronic phase is not obvious.

### 2.2. Complexity measures

Five complexity metrics, including ApEn, SampEn, FuzzEn, PE, and KC, have been computed to quantify the dynamic changes of EEG signals during different stages of epileptogenesis. A brief introduction to these metrics is given in this section.

#### 2.2.1. Approximate entropy

Approximate Entropy (ApEn) was proposed by Pincus et al. (1991) from the perspective of measuring the complexity of signal. It is a non-linear dynamic measure that quantifies the incidence of new information in the time series (Pincus et al., 1991). The higher the probability of a new pattern being generated in this time series, the higher the complexity of the sequence and the higher the corresponding ApEn value.

The calculation of ApEn is calculating the degree of self-similarity of a time series, that is, the difference between the probability of mutual approximation of m points adjacent to the sequence and the probability of mutual approximation of *m*+1 points. Compared with the statistical characteristics such as mean and variance, ApEn can better reflect the characteristics of signal sequence in structural distribution.

#### 2.2.2. Sample entropy

In order to reduce the estimation bias in the calculation of ApEn by comparing it to its own data segment, Sample Entropy (SampEn) was proposed by Richman and Moorman (2000). Different from ApEn, SampEn eliminates self-matches in the algorithm and computes the difference of logarithms of the probabilities. Therefore, SampEn is more accurate, more consistent, and not sensitive to the missing values.

#### 2.2.3. Permutation entropy

Proposed by Bandt and Pompe (2002), Permutation Entropy (PE) provides a quantification measure of the complexity of a time series by capturing the order relations between reconstructed subsequences. Computed from the extracted probability distribution of the ordinal patterns (Henry and Judge, 2019), the value of PE may account for the temporal ordering structure (time causality) of a given time series. The PE approach is robust to noise, computationally efficient, and invariant with respect to non-linear monotonic transformations of the data.

#### 2.2.4. Fuzzy entropy

Inspired by the concept of fuzzy set (Zadeh et al., 1996), Chen et al. (2007) proposed a new measure of complexity for time series in 2007, called Fuzzy Entropy (FuzzEn). Modified from ApEn and SampEn, but unlike them, FuzzEn measures the similarity of two vectors based on the idea of “fuzzy.” That is, the similarity is no longer 1 or 0 determined by a single threshold but a fuzzy membership function, thereby blurring the similarity measure.

#### 2.2.5. Kolmogorov complexity

As an early complexity measure, Kolmogorov Complexity (KC) was first proposed by Solomonoff (1960) and then developed by Chaitin (1977). According to Li and Vitányi (2008), for a given string or sequence, KC is defined as the size of the smallest program that is needed to generate that string. It was also known as “algorithmic complexity,” “Kolmogorov-Chaitin complexity,” “shortest program length,” etc. Unlike Shannon's information theory, KC is a measure of randomness or irregularity of individual objects rather than the average information of a random source.

### 2.3. Classification

In order to integrate all these complexity metrics at different channels, in this section, a GCNN-based classification framework is proposed and implemented to automatically identify and detect the acute and chronic stages of epilepsy.

#### 2.3.1. Graph convolutional neural network (GCNN)

**Figure 2:**
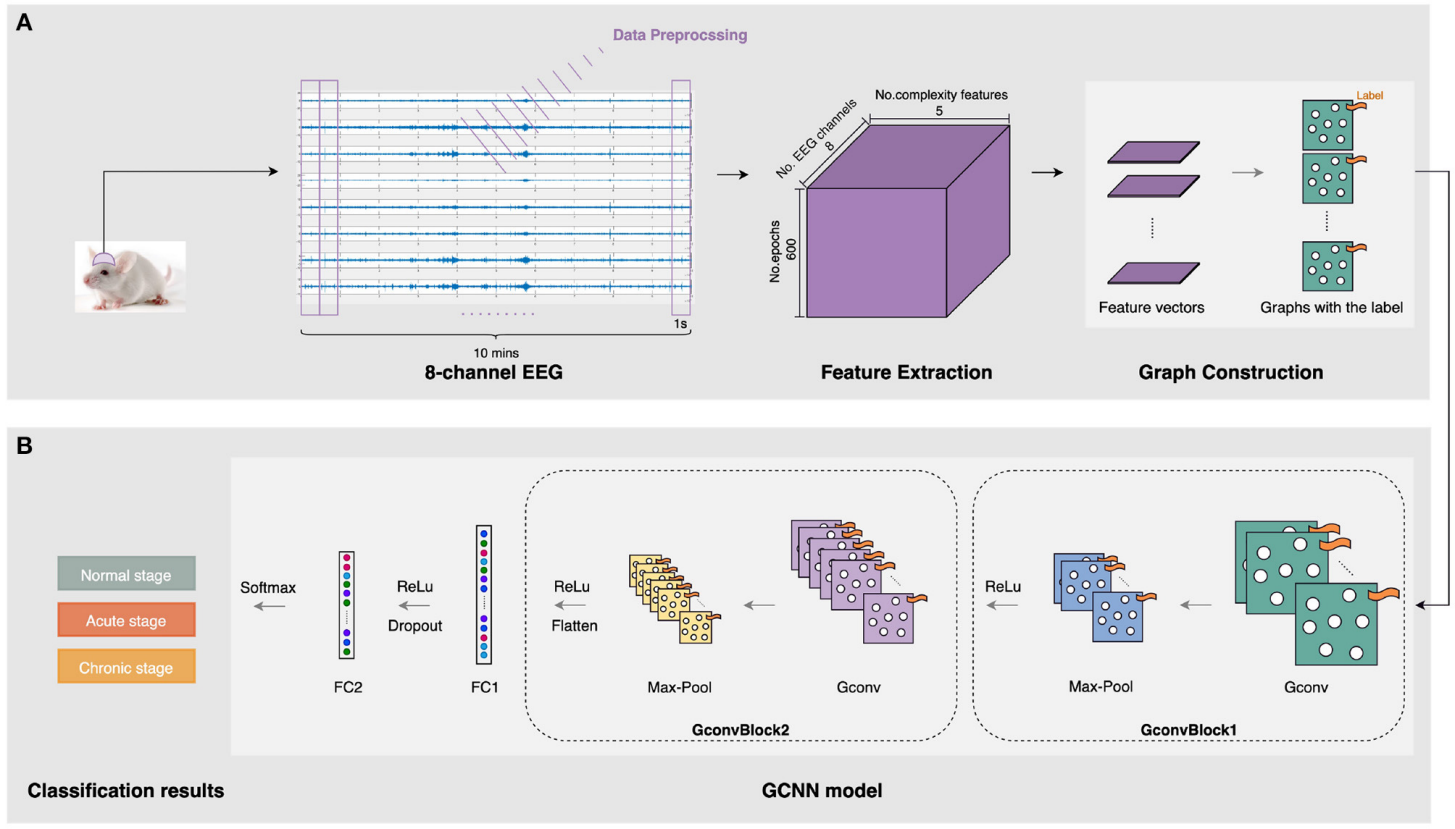
The architecture of the three-stage epileptic detection using complexity-GCNN classifier. **(A)** Graph construction for five complexity measures at eight channels. **(B)** epileptic detection GCNN classification model.

Our automatic epileptic detection system is built on GCNN proposed by Defferrard et al. (2016). GCNN is an extension framework that combines classical convolutional neural networks (CNN) and spectrum theory. Three main steps are involved to generalize CNNs to graphs, including designing the localized convolutional filters on graphs, clustering the similar vertices, and transforming spatial resolution for higher filter resolution (Defferrard et al., 2016). Thus, in addition to retaining the advantages of CNN, GCNN can deal with homogeneous and heterogeneous data (Such et al., 2017). In particular, it is capable of extracting features from unstructured data, such as graph representations, by performing convolutions on graph signals (Raeisi et al., 2022). Meanwhile, using graph as the input, GCNN provides a useful tool for processing signals from multiple channels simultaneously. Figure 2 shows a flow diagram of this automatic epileptic detection system for distinguishing EEG signals during the acute or chronic stage of epilepsy from normal.

##### 2.3.1.1. Graph construction

As presented in Figure 2A, the inputs of our GCNN classifier are constructed on graphs with complexity measures. After collecting and preprocessing the 10-min 8-channel EEG as mentioned in Section 2.1, five complexity characteristics were extracted from each 1s-epoch EEG of each channel. To construct graphs, the sets of features are organized as a matrix. In particular, each feature matrix for a 1s-epoch EEG has eight rows and five columns, representing five extracted features at eight channels. Then, graphs representing five kinds of complexity at eight channels were generated and labeled with their specific stage (i.e., normal/acute/chronic). In this case, we notice that the connectivity pattern between channels may exist some kind of similarity in three stages of epilepsy. Therefore, to reduce potential interference due to this continuity between the three different stages, we construct each complete graph with eight nodes and all edges equal to 1, as the input to GCNN.

##### 2.3.1.2. GCNN classification model

To achieve epileptic detection tasks, the constructed graphs were inputted to the classifier for training and validation to find the best GCNN model in identifying the specific stages (i.e., normal/acute/chronic) of current EEG fragments. As presented in Figure 2B, this GCNN network comprises two graph convolution blocks, two fully connected (FC) layers, and a softmax output layer. Each convolution block consists of a graph convolution layer, a max-pooling layer, and a Rectified Linear Unit (ReLU) active function. Specifically, the purpose of the convolution layer is to capture the features from the input graphs and learn the features that would be useful for the classification tasks. The max-pooling layer is a down-sample operation, which reduces the computation and avoids overfitting by decreasing the number of parameters to learn. Afterward, the ReLu layer will replace the input with zero if it is negative; otherwise, it will retain the original value. It is expressed by:


(1) $$\begin{array}{l} ReLu\left(x\right)=max\left(0,x\right), \end{array}$$

After a repeated graph convolution block, two FC layers followed. In particular, between these two FC layers, a ReLu layer was used, and a regularization technique called dropout was applied to avoid overfitting. Finally, the softmax activation function was used for three-stage epileptic detection tasks to obtain the result. The detailed configuration of this GCNN classification model is shown in Table 2.

**Table 2:** The configuration of the GCNN-based classifier.

	**Layer**	**Output size (Tensor)**
Input		[6^*^, 1, 8, 5]
GconvBlock1	Graph convolution	
	Pool	
	ReLU	[6, 10, 4, 3]
GconvBlock2	Graph convolution	
	Pool	
	ReLU	[6, 20, 2, 1]
	Flatten	[6, 40]
FC1	Fully connected	
	ReLU	[6, 15]
	Dropout	[6, 15]
FC2	Fully connected	[6, 3]
Prediction	Softmax	[6, 3]

^*^The batch size of training is six and the result will contain six training units.

#### 2.3.2. Evaluation metrics

Three typical assessment methods: confusion matrix, accuracy and F1 score are employed to evaluate the classification performance of the GCNN model constructed on complexity measures.

##### 2.3.2.1. Confusion matrix

It is a 3 × 3 matrix that tells us the rate of true positives and false positives when the sampled signal is from normal, acute, and chronic stages, respectively.

##### 2.3.2.2. Accuracy

The overall accuracy is a classifier's ability to correctly predict the classes and is defined as:


(2) $$\begin{array}{l} Accuracy=\frac{Correct\;Predictions}{Total\;Predictions}\times 100\%. \end{array}$$

##### 2.3.2.3. F1 score

The F1 score refers to a balanced measure between two other metrics: precision and recall, where precision is the ability of the classifier to identify the positive class with accuracy, and recall is the ability of a model to predict each of the positive observations within a data set correctly. It is expressed as:


(3) $$\begin{array}{l} F1\;score=\frac{2\times Precision\times Recall}{Precision+Recall}\times 100\%. \end{array}$$

## 3. Results and discussion

This section demonstrates the main results of EEG complexity analysis and three-stage epileptic detection.

The procedures of EEG processing and feature extraction were carried out using MATLAB R2022a. Statistical analyses were performed using SPSS 25.0, and the GCNN-based three-stage epileptic classification was conducted using Python 3.9.12.

During data processing, each 10-min EEG recording sample with 600,000 data points was divided into non-overlapping 1s epochs, resulting in 600 epochs and 1,000 data points in each epoch. Then, EEG signals were decomposed by wavelet transform based on the Haar wavelet and extracted a specific frequency band spanning 250–500 Hz (Fast Ripples). Following the data pre-processing, five complexity measures, including ApEn, SampEn, PE, FuzzEn, and KC, are calculated on each EEG epoch of the eight channels for further analysis.

### 3.1. Dynamic changes in complexity

**Figure 3:**
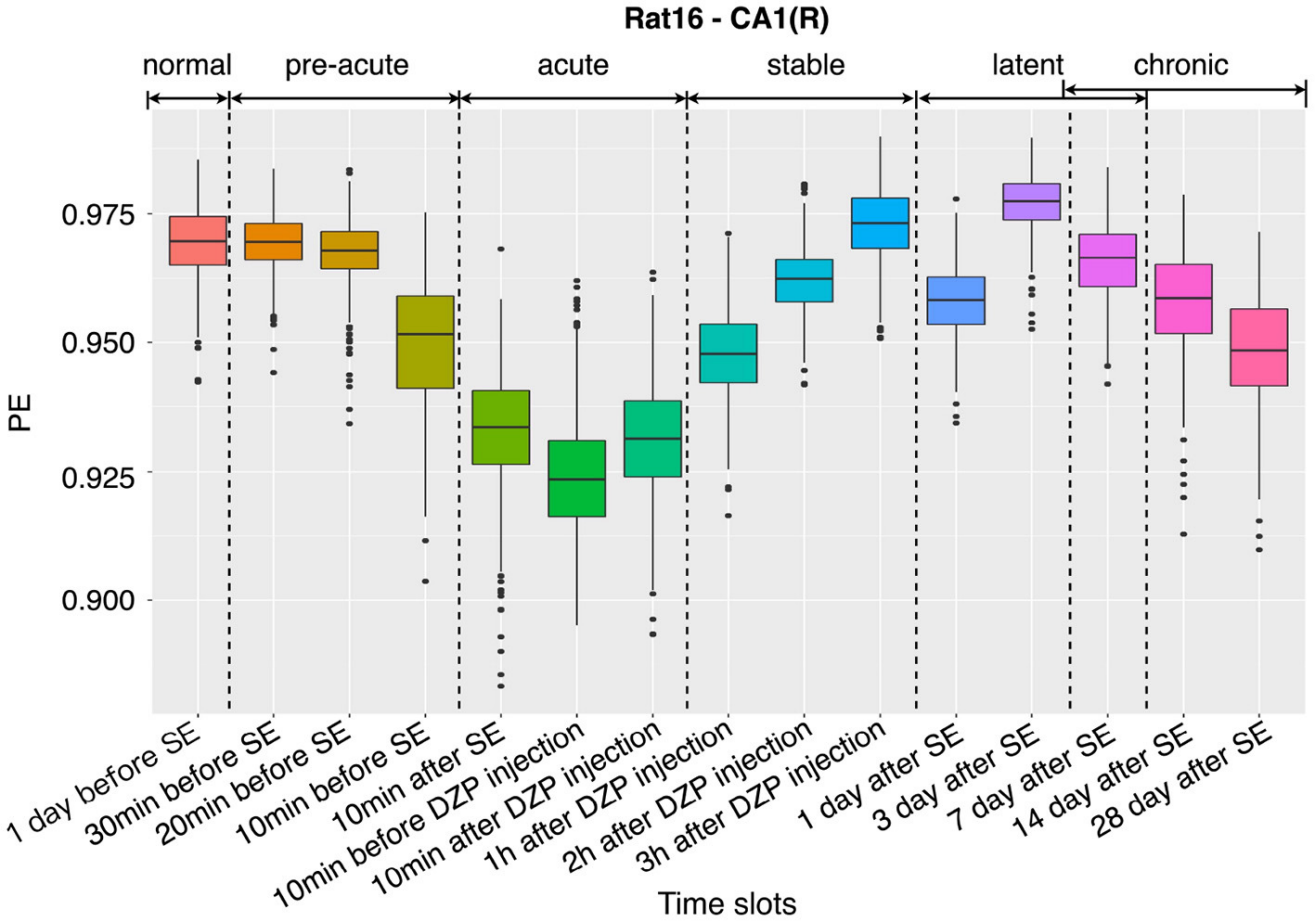
Dynamic changes of the complexity at 15 successive time slots of PILO modeling.

To demonstrate the dynamic changes of the complexity for all stages mentioned in Section 2.1, a boxplot of the PE distributions at 15 successive stages of the channel CA1(L) of representative rat (no.16) is given in Figure 3. It was found that in the normal period (1 day before SE), the PE values are at a relatively high level, and the EEG shows a large randomness. The complexity starts to drop 30 min before SE, then continues to fall sharply until the DZP is injected. The decreasing of the complexity suggests that with the onset of epilepsy, EEG gradually presents some regular rhythms, which reduces the complexity. Afterward, from 10 min after DZP injection, PE values continue rising and recover to normal by 3 h after DZP injection. However, after the effect of DZP subsides, it is found that the values of PE begin to decline to a certain extent in the chronic stage. This indicates the appearance of SRSs. Using PE as a representative of EEG complexity clearly shows the dynamic changes of the brain's electrical activity before and after SE, in the process of seizure and DZP injection, and the chronic phase (Figure 3).

### 3.2. Statistical significance

EEG Complexity metrics at normal, acute, and chronic stages were compared through one-way ANOVA. The *F*-test statistics and the two-tailed *p*-values were presented in Table 3. Tukey's test was performed for pairwise comparison for the complexity between any two of the stages, and the mean differences (*p*-values) for normal and acute stages, normal and chronic stages were also given in Table 3. In this part, three 10-min EEG recordings, including “1 day before SE,” “10 min before DZP injection,” and “28 days after SE” were selected to represent normal, acute epilepsy, and chronic epilepsy, respectively. Each 10-min EEG recording was divided into 20 equal-length epochs. So, the number of each computed complexity measure for normal, acute, and chronic groups in one-way ANOVA is 160, including epochs from eight channels.

**Table 3:** Results of one-way ANOVA for distinguishing normal, acute, and chronic phases.

**Complexity measure**	**Mean difference in multiple comparisons**		***F*-test**	***p*-value**
	**Normal-acute (*p*-value)**	**Normal-chronic (*p*-value)**		
ApEn	0.7710 (5.1e-9)	0.0961 (5.4e-9)	1660.3	1.3e-215
SampEn	1.4203 (5.1e-9)	0.0858 (8.8e-8)	5502.9	0
PE	0.0310 (5.1e-9)	0.0200 (5.1e-9)	302.4	1.5e-85
FuzzEn	0.6199 (5.1e-9)	0.1011 (5.1e-9)	1711.6	2.2e-218
KC	0.1014 (5.1e-9)	0.0125 (8.2e-9)	1493.2	4.5e-206

Through the results of one-way ANOVA, we found that using complexity as a feature can well reflect the differences between normal, acute, and chronic phases. Regardless of the type of complexity, the *p*-values of the *F*-tests are close to zero. In the pairwise comparisons using Tukey *post-hoc* testing, there is also a significant difference in complexity between normal and acute phases, as well as between normal and chronic phases, with *p*-values all below 10^−7^. These results indicate that complexity measures are beneficial features in distinguishing different stages of epilepsy.

**Figure 4:**
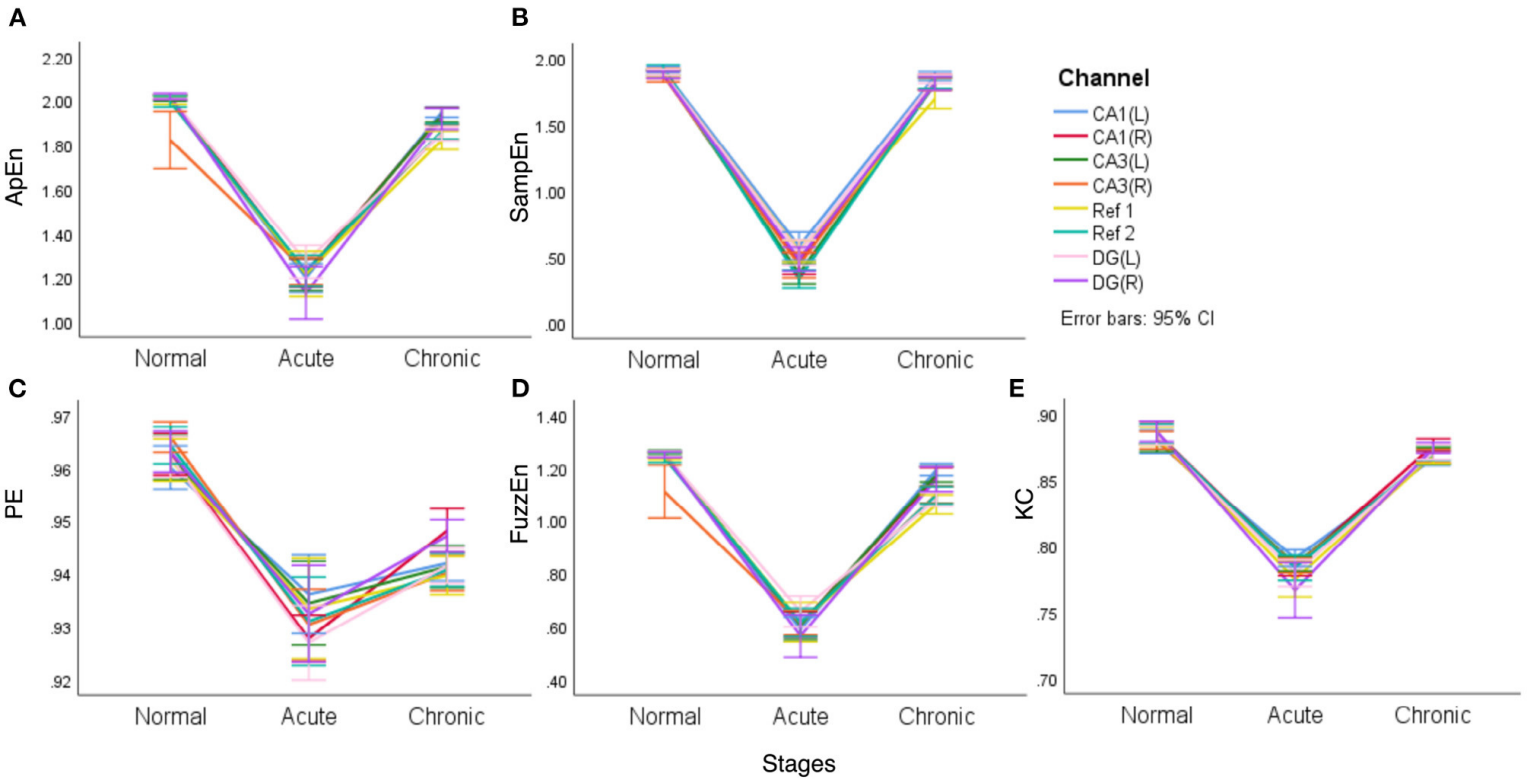
The line charts with error bars (95% CI) of each of the complexity measures in three epileptic stages for representative rat (no.16) **(A–E)**.

In fact, the difference between normal and chronic stages is rarely mentioned in literature. Song et al. (2016) tried to detect and quantify different phases of epileptogenesis by implementing average and peak spectral power of high-frequency oscillations (HFOs). They successfully found the dynamic changes between the acute and normal stages but failed to show statistical significance for differences between the chronic and normal stages using spectral power, the characteristic based on linear theories. Meanwhile, line charts of means and their 95% confidence intervals (CI) are presented to visualize the differences for all the five complexity measures in acute, normal, and chronic phases (Figure 4). Lines with eight colors represent eight EEG signal channels, including two reference channels (Ref 1 and Ref 2).

It is clear from Figure 4 that different complexity measures reflect similar laws, that is, the mean complexity of EEG is at a relatively high value in the normal period, while in the acute phase of epilepsy, the mean complexity has a significant decline, which confirms that during epilepsy, EEG will continue to appear some particular waveforms and become regular. In the chronic period, entropy will rise again, even returning to a level close to the normal phase but slightly lower than the normal phase. In particular, for PE, the gap between the normal and chronic phases is relatively apparent. Another noteworthy point is that two reference channels (Ref 1 and Ref 2) are also included in this comparison. However, it is interesting to see from the line charts listed in Figure 4 that these two reference channels (Ref 1 and Ref 2) express similar complexity during the main stages of PILO modeling.

### 3.3. Classification performance

**Figure 5:**
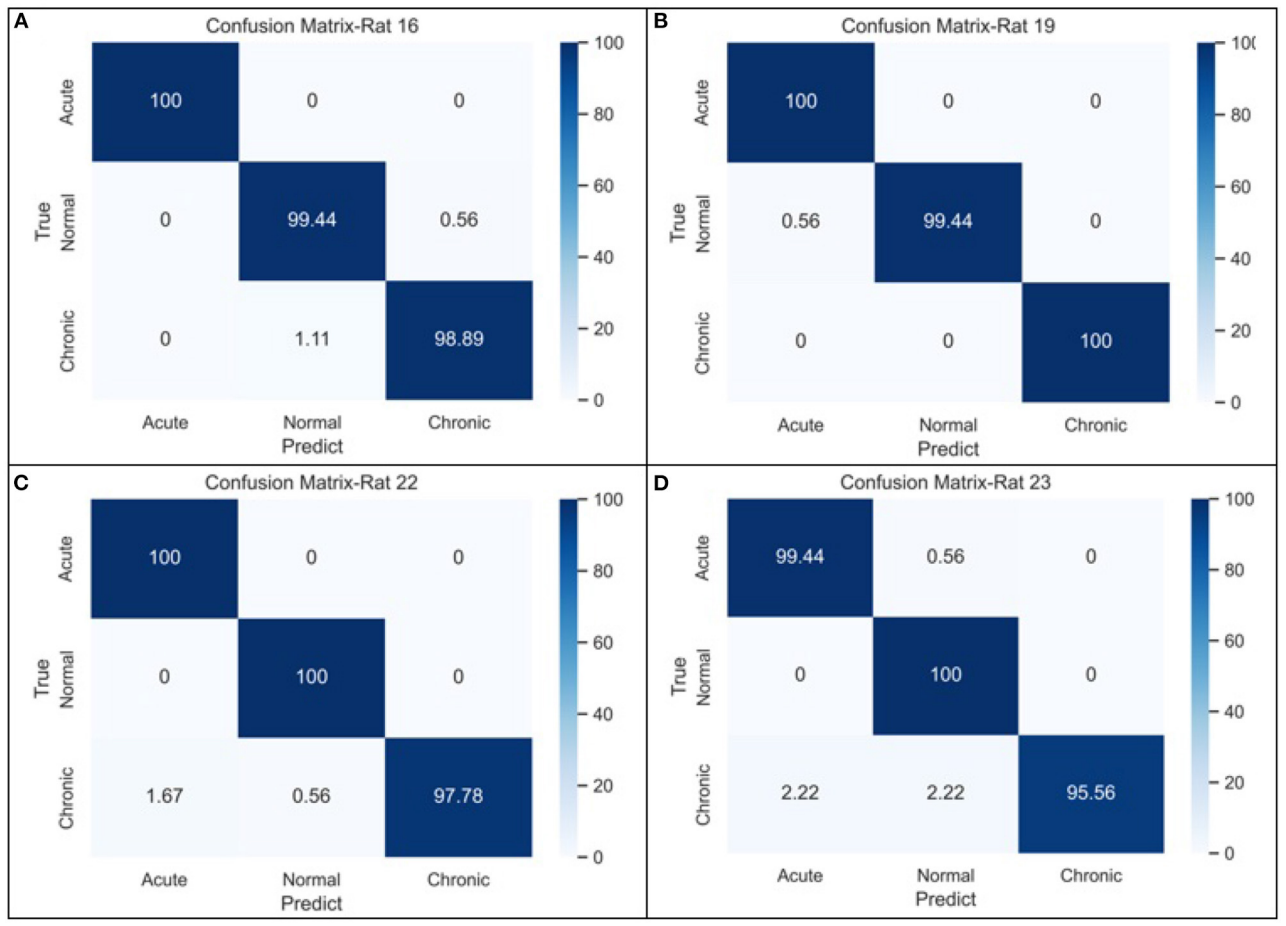
The confusion matrices of GCNN-based classifier for three-stage epileptic detection based on **(A)** rat no.16, **(B)** rat no.19, **(C)** rat no.22, and **(D)** rat no.23, respectively.

To evaluate the performance of complexity indicators in classifying the normal, acute, and chronic stages of epilepsy, we conduct GCNN-based classification with hyperparameter settings listed in Table 4 for each individual rat, and across all rats. The data was split into training, validation and testing sets, with a 50–20–30% partition. Figure 5 includes four confusion matrices obtained for four rats, where the detection rates of the three phases are calculated. Other useful evaluation indicators of model classification such as accuracy and F1 score are also listed in Table 5.

**Table 4:** The hyperparameter settings of the GCNN-based classifier for classification.

**Hyperparameter**	**Values**	
	**For individual subject**	**Across all subjects**
Learning rate	0.001	0.001
Epochs	3	50
Batch size (Train)	6	6
Batch size (Test)	2,175	540
Momentum	0.5	0.5
Log interval	10	10
Activation function	ReLU	ReLU

**Table 5:** Classification performance of GCNN based on complexity measures.

**Subject**	**Accuracy (%)**	**F1 score (%)**		
		**Normal**	**Acute**	**Chronic**
Rat no.16	0.9944	1.0000	0.9917	0.9916
Rat no.19	0.9981	0.9972	0.9972	1.0000
Rat no.22	0.9926	0.9917	0.9972	0.9888
Rat no.23	0.9833	0.9862	0.9863	0.9773
Combined	0.8782	0.9725	0.8603	0.7927

From the confusion matrices shown in Figure 5, the probability of being detected (i.e., sensitivity) for acute and normal phases is relatively high, reaching between 99.45 and 100%, while the detection rate of chronic phase is slightly lower, but still more than 95%. The classification performance across all subjects is shown in the last row of Table 5. It can be seen that when the measures of the four rats were merged, the effectiveness of classification decreased considerably due to the heterogeneity among individual rats.

**Figure 6:**
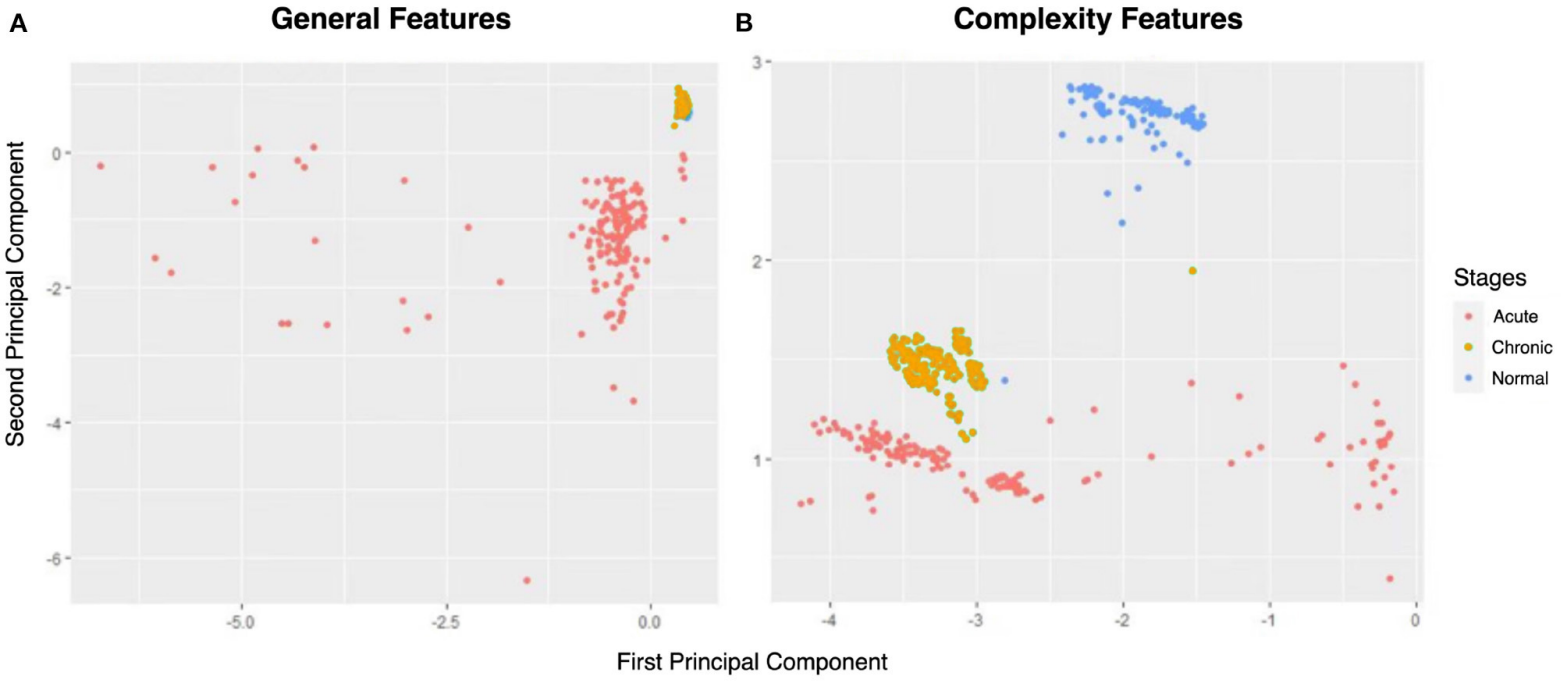
The principal component analysis (PCA) score plots of **(A)** general features and **(B)** complexity features.

To demonstrate the superiority of complexity metrics in differentiating chronic phases of epilepsy, we calculated two sets of EEG characteristics: one includes five complexity measures, and another has five general features: mean, variance, maximum, minimum, and skewness. Taking representative rat (no.16) as an example, the principal component (PC) method is applied to the two normalized five-dimensional characteristic data matrices to compress them to two-dimensional metrics. Figure 6 are 2-PC plots obtained from these two sets of features.

From Figure 6, the normal and acute phases can be well distinguished under either set of features. However, general indicators and complexity measures differ in their ability to distinguish normal and chronic phases. As shown in Figure 6A, there is a significant overlap between the yellow (i.e., chronic phase) and blue points (i.e., normal phase), so the general indicators mix these two phases. Nevertheless, the points of normal and chronic phases can be easily recognized using complexity measures (Figure 6B). Thus, the comparison in Figure 6 gives us a preliminary impression that complexity measurement can effectively identify the chronic phase of epilepsy.

## 4. Conclusion

In this paper, the differences in EEG between normal and chronic phases of epilepsy for rats were studied in depth for the first time. By calculating five commonly used complexity measures: ApEn, SampEn, PE, FuzzEn, and KC, the dynamic changes in brain waves during seizures can be perfectly displayed. Results of one-way ANOVA and PCA score plots show that complexity features can well reflect the differences between normal, acute, and chronic phases with extremely small *p*-values. In particular, among with these complexity metrics, PE exhibits the greatest discrepancy between normal and chronic stages. In order to integrate five complexity measures at eight channels, an automatic epileptic detection system via GCNN is developed. Our model reaches high performance in epilepsy detection that the recognition rate of each individual rat can achieve more than 98%, even 100%, including normal and chronic stages. In our case study, a comparison between modeling based on each individual subject and modeling across all subjects highlighted the non-negligible heterogeneity among individual rats. Modeling across all subjects may inadequately account for these individual differences, thus diminishing the model's fit to individual data. In contrast, modeling based on each individual subject can provide highly personalized models for each individual, significantly enhancing model accuracy, especially when the chronic phase is considered. This underscores the necessity of employing modeling based on each individual subject for personalized treatment recommendations in practical epilepsy management, ensuring better alignment with patients' unique needs.

While the above experiments yielded promising results in the classification of three epilepsy stages, our investigation was limited to the effectiveness of this framework solely in rat data and for just one type of epilepsy. In future work, we intend to extend the application of this framework to human EEG datasets. Concurrently, we will make adjustments to both graph representations and model parameters to elucidate the distinct characteristics of human EEG data, thus enhancing the model's generalization capabilities.

## Data availability statement

The original contributions presented in the study are included in the article/supplementary material, further inquiries can be directed to the corresponding author.

## Author contributions

SZ proposed the work, analyzed the data, and wrote the manuscript. XZ did the main computation work. PS did the experiment and generated the data. YH interpreted the results and provided guidance. XG analyzed the data and revised the manuscript. XP conducted the whole work. All authors contributed to the article and approved the submitted version.

## References

[B1] Arunkumar N. Ramkumar K. Venkatraman V. Abdulhay E. Fernandes S. L. Kadry S.. (2017). Classification of focal and non-focal EEG using entropies. Pattern Recogn. Lett. 94, 112–117. 10.1016/j.patrec.2017.05.007

[B2] Bandt C. Pompe B. (2002). Permutation entropy: a natural complexity measure for time series. Phys. Rev. Lett. 88, 174102. 10.1103/PhysRevLett.88.174102 12005759

[B3] Boonyakitanont P. Lek-Uthai A. Chomtho K. Songsiri J. (2020). A review of feature extraction and performance evaluation in epileptic seizure detection using EEG. Biomed. Signal Process. Control 57, 101702. 10.1016/j.bspc.2019.101702

[B4] Bullmore E. Sporns O. (2009). Complex brain networks: graph theoretical analysis of structural and functional systems. Nat. Rev. Neurosci. 10, 186–198. 10.1038/nrn2575 19190637

[B5] Chaitin G. J. (1977). Algorithmic information theory. IBM J. Res. Dev. 21, 350359. 10.1147/rd.214.0350

[B6] Chen W. Wang Z. Xie H. Yu W. (2007). Characterization of surface EMG signal based on fuzzy entropy. IEEE Trans. Neural Syst. Rehabil. Eng. 15, 266–272. 10.1109/TNSRE.2007.897025 17601197

[B7] Covert I. C. Krishnan B. Najm I. Zhan J. Shore M. Hixson J.. (2019). “Temporal graph convolutional networks for automatic seizure detection,” in Proceedings of Machine Learning Research 106 (PMLR), 160–180.37497802

[B8] Craley J. Jouny C. Johnson E. Hsu D. Ahmed R. Venkataraman A. (2022). Automated seizure activity tracking and onset zone localization from scalp EEG using deep neural networks. PLoS ONE 17, e0264537. 10.1371/journal.pone.0264537 35226686 PMC8884583

[B9] Defferrard M. Bresson X. Vandergheynst P. (2016). “Convolutional neural networks on graphs with fast localized spectral filtering,” in Proceedings of the 30th International Conference on Neural Information Processing Systems, NIPS'16 (Red Hook, NY: Curran Associates Inc.), 3844–3852

[B10] Faust O. Acharya U. R. Min L. C. Sputh B. H. (2010). Automatic identification of epileptic and background EEG signals using frequency domain parameters. Int. J. Neural Syst. 20, 159–176. 10.1142/S0129065710002334 20411598

[B11] Fisher R. S. Boas W. V. E. Blume W. Elger C. Genton P. Lee P.. (2005). Epileptic seizures and epilepsy: definitions proposed by the international league against epilepsy (ILAE) and the international bureau for epilepsy (IBE). Epilepsia 46, 470–472. 10.1111/j.0013-9580.2005.66104.x 15816939

[B12] Henry M. Judge G. (2019). Permutation entropy and information recovery in nonlinear dynamic economic time series. Econometrics 7, 10. 10.3390/econometrics7010010

[B13] Hou Y. Jia S. Lun X. Hao Z. Shi Y. Li Y.. (2022). GCNs-Net: a graph convolutional neural network approach for decoding time-resolved EEG motor imagery signals. IEEE Trans. Neural Netw. Learn. Syst. 1–12. 10.1109/TNNLS.2022.3202569 36099220

[B14] Jia Z. Lin Y. Wang J. Zhou R. Ning X. He Y.. (2020). “Graphsleepnet: adaptive spatial-temporal graph convolutional networks for sleep stage classification,” in Proceedings of the Twenty-Ninth International Joint Conference on Artificial Intelligence (IJCAI-20), Vol. 2021, 1324–1330. 10.24963/ijcai.2020/184

[B15] Karlócai M. R. Tóth K. Watanabe M. Ledent C. Juhász G. Freund T. F.. (2011). Redistribution of cb1 cannabinoid receptors in the acute and chronic phases of pilocarpine-induced epilepsy. PLoS ONE 6, e27196. 10.1371/journal.pone.0027196 22076136 PMC3208595

[B16] Li G. Jung J. J. (2021). Seizure detection from multi-channel EEG using entropy-based dynamic graph embedding. Artif. Intell. Med. 122, 102201. 10.1016/j.artmed.2021.102201 34823838

[B17] Li M. Chen W. Zhang T. (2017). Automatic epileptic EEG detection using DT-CWT-based non-linear features. Biomed. Signal Process. Control 34, 114–125. 10.1016/j.bspc.2017.01.010

[B18] Li M. Vitányi P. (2008). An Introduction to Kolmogorov Complexity and Its Applications, Vol. 3. New York, NY: Springer.

[B19] Lian Q. Qi Y. Pan G. Wang Y. (2020). Learning graph in graph convolutional neural networks for robust seizure prediction. J. Neural Eng. 17, 035004. 10.1088/1741-2552/ab909d 32375134

[B20] Liang Z. Wang Y. Sun X. Li D. Voss L. J. Sleigh J. W.. (2015). EEG entropy measures in anesthesia. Front. Comput. Neurosci. 9, 16. 10.3389/fncom.2015.00016 25741277 PMC4332344

[B21] Natarajan K. Acharya U R. Alias F. Tiboleng T. Puthusserypady S. K.. (2004). Nonlinear analysis of EEG signals at different mental states. Biomed. Eng. Online 3, 1–11. 10.1186/1475-925X-3-7 15023233 PMC400247

[B22] Pincus S. M. Gladstone I. M. Ehrenkranz R. A. (1991). A regularity statistic for medical data analysis. J. Clin. Monit. 7, 335–345. 10.1007/BF01619355 1744678

[B23] Raeisi K. Khazaei M. Croce P. Tamburro G. Comani S. Zappasodi F. (2022). A graph convolutional neural network for the automated detection of seizures in the neonatal EEG. Comput. Methods Prog. Biomed. 2022, 106950. 10.1016/j.cmpb.2022.106950 35717740

[B24] Richman J. S. Moorman J. R. (2000). Physiological time-series analysis using approximate entropy and sample entropy. Am. J. Physiol. Heart Circul. Physiol. 278, H2039–H2049. 10.1152/ajpheart.2000.278.6.H2039 10843903

[B25] Saxena S. Li S. (2017). Defeating epilepsy: a global public health commitment. Epileps. Open 2, 153–155. 10.1002/epi4.12010 29588944 PMC5719859

[B26] Sharma R. Pachori R. B. Acharya U. R. (2014). Application of entropy measures on intrinsic mode functions for the automated identification of focal electroencephalogram signals. Entropy 17, 669–691. 10.3390/e17020669

[B27] Sharmila A. Geethanjali P. (2018). Effect of filtering with time domain features for the detection of epileptic seizure from EEG signals. J. Med. Eng. Technol. 42, 217–227. 10.1080/03091902.2018.1464075 29798699

[B28] Solomonoff R. J. (1960). A Preliminary Report on a General Theory of Inductive Inference. Citeseer.

[B29] Song P. Xiang J. Jiang L. Chen H. Liu B. Hu Y. (2016). Dynamic changes in spectral and spatial signatures of high frequency oscillations in rat hippocampi during epileptogenesis in acute and chronic stages. Front. Neurol. 7, 204. 10.3389/fneur.2016.00204 27965619 PMC5124575

[B30] Song T. Zheng W. Song P. Cui Z. (2018). EEG emotion recognition using dynamical graph convolutional neural networks. IEEE Trans. Affect. Comput. 11, 532–541. 10.1109/TAFFC.2018.2817622

[B31] Srinivasan V. Eswaran C. Sriram N. (2005). Artificial neural network based epileptic detection using time-domain and frequency-domain features. J. Med. Syst. 29, 647–660. 10.1007/s10916-005-6133-1 16235818

[B32] Such F. P. Sah S. Dominguez M. A. Pillai S. Zhang C. Michael A.. (2017). Robust spatial filtering with graph convolutional neural networks. IEEE J. Select. Top. Signal Process. 11, 884–896. 10.1109/JSTSP.2017.272698137669194

[B33] Tzallas A. T. Tsipouras M. G. Fotiadis D. I. (2009). Epileptic seizure detection in EEGs using time-frequency analysis. IEEE transactions on information technology in biomedicine, 13, 703–710. 10.1109/TITB.2009.2017939 19304486

[B34] Wagh N. Varatharajah Y. (2020). “EEG-GCNN: augmenting electroencephalogram-based neurological disease diagnosis using a domain-guided graph convolutional neural network,” in Proceedings of Machine Learning Research 136 (PMLR), 367–378.

[B35] Wang L. Xue W. Li Y. Luo M. Huang J. Cui W.. (2017). Automatic epileptic seizure detection in EEG signals using multi-domain feature extraction and nonlinear analysis. Entropy 19, 222. 10.3390/e1906022232078551

[B36] Wei Z. Zou J. Zhang J. Xu J. (2019). Automatic epileptic EEG detection using convolutional neural network with improvements in time-domain. Biomed. Signal Process. Control 53, 101551. 10.1016/j.bspc.2019.04.02835077027

[B37] Wen T. Zhang Z. (2017). Effective and extensible feature extraction method using genetic algorithm-based frequency-domain feature search for epileptic EEG multiclassification. Medicine 96, 6879. 10.1097/MD.0000000000006879 28489789 PMC5428623

[B38] Xiang J. Li C. Li H. Cao R. Wang B. Han X.. (2015). The detection of epileptic seizure signals based on fuzzy entropy. J. Neurosci. Methods 243, 18–25. 10.1016/j.jneumeth.2015.01.015 25614384

[B39] Yuan Q. Zhou W. Li S. Cai D. (2011). Epileptic EEG classification based on extreme learning machine and nonlinear features. Epilepsy Res. 96, 29–38. 10.1016/j.eplepsyres.2011.04.013 21616643

[B40] Zadeh L. A. Klir G. J. Yuan B. (1996). Fuzzy Sets, Fuzzy Logic, and Fuzzy Systems: Selected Papers, Vol. 6. World Scientific. 10.1142/2895

[B41] Zhang T. Wang X. Xu X. Chen C. P. (2019). GCB-Net: graph convolutional broad network and its application in emotion recognition. IEEE Trans. Affect. Comput. 13, 379–388. 10.1109/TAFFC.2019.2937768

